# Knowledge, Attitude and Practices of Labor Analgesia amongst healthcare workers and patients: a single center cross sectional study

**DOI:** 10.12669/pjms.36.ICON-Suppl.1715

**Published:** 2020-01

**Authors:** Maher Ali, Syed Farjad Sultan, Anil Kumar, Nida Ghouri

**Affiliations:** 1 Dr. Maher Ali, MBBS. Specialist Anaesthesiologist, Department of Anaesthesia, Civil Hospital Badin, Indus Health Network, Badin, Pakistan; 2 Dr. Syed Farjad Sultan, PhD. Consultant Anaesthetist Department of Anaesthesia, Surgical Intensive Care and Pain Management. Ruth KM Pfau, Civil Hospital Karachi, Karachi - Pakistan; 3 Dr. Anil Kumar, MBBS. Specialist Anaesthesiologist, Department of Anaesthesia, Intensive care and Pain Medicine, Indus Hospital Research Centre, The Indus Hospital, Karachi, Pakistan; 4 Nida Ghouri, Research Associate, Indus Hospital Research Centre, The Indus Hospital, Karachi, Pakistan

**Keywords:** Epidural, Labor analgesia, Obstetrics, Regional anaesthesia

## Abstract

**Background and Objective:**

Childbirth ranks amongst the most painful experiences a woman has to endure. In developing countries issues related to awareness, acceptability and availability of analgesia exist. This study aims to assess the knowledge, attitude and practices for labor analgesia amongst healthcare workers and patients.

**Methods:**

We performed a cross-sectional study conducted at Shaikh Saeed Memorial Campus of The Indus Hospital (TIH), Karachi. The study was performed in November 2016. Two surveys were conducted for this study; one for healthcare workers with patient interaction and second on patients attending their first antenatal clinic. The participants were asked to complete a survey following consent.

**Results:**

A total of 71 healthcare workers and 1005 patients participated in the study. Among healthcare workers, 43.7% felt that pain relief should be administered, 14.1% believed analgesia had adverse effects on fetus and 11.3% reported increased risk of cesarean section. Results from patients showed 27.9% were aware of labor analgesia and when informed, 85.2% were willing to have labor analgesia. In 14.1% of patients, labor analgesia was unsupported as they stated being a mother meant to endure pain.

**Conclusion:**

There is a wide gap between knowledge and practice of labor analgesia. Healthcare workers have a role in educating women, to be advocates for labor analgesia and to educate patients timely for this service.

## INTRODUCTION

Childbirth is one of the most painful experiences of a woman’s life. A lack of knowledge regarding the birth process and pain during labor and delivery can influence a woman’s attitude towards childbirth. The American Society of Anaesthesiologists, American College of Obstetricians and Gynecologists and National Institute of Clinical Excellence in the United Kingdom recommend provision of education to expectant mothers on the options and availability of effective pain relief for labor.^1^-^3^

Labor analgesia in high income countries focuses on methods and complications, while in low and middle income countries (LMICs) awareness and availability of services are of concern^4^. Culture, knowledge, ethnic group, age, education and socio-economic status may have a role in attitude towards pain relief in labor.^4^,^5^

A number of studies have been conducted worldwide, specifically in Africa to determine patient factors.^4^-^6^ However, the attitudes of healthcare staff regarding labor analgesia has not been established in LMICs and addressing pain relief is often neglected.^7^

This study aimed to assess the level of awareness, knowledge and attitude of healthcare staff and patients on their first visit to the antenatal clinic towards labor pain and labor analgesia.

## METHODS

We performed a cross-sectional study after obtaining the necessary institutional and ethical approvals (IRD_IRB_2016_10_002). This study was conducted at Shaikh Saeed Memorial Campus of The Indus Hospital (TIH), Karachi during November 2016. TIH is a free-of-cost hospital located in Korangi, serving many low-income communities in Karachi.

All staff members with patient contact (healthcare workers, HCW) and all patients attending the antenatal clinic on their first visit, in November 2016, were invited to participate. Both groups of participants completed the same questionnaire as part of this survey.

The questionnaire was adopted from a study performed by Nabukenya et al.^6^ and translated into the local language. The first section examined demographics (age, education level, parity and previous delivery). The second section consisted of questions to assess knowledge and options of labor analgesia, sources of information and perception on labor analgesia. The last section of the study was related to labor analgesia, gave knowledge of labor analgesia to those patients who did not know of it and then asked for willingness to receive labor analgesia.

On average the translated survey took 10 minutes. Each question examined a single idea (no questions contained “and”) and no questions were phrased in a negative form. Answers were in yes/no/neutral or pain scores on a scale of 0 to 10. The questions were asked via a trained investigator (First Author).

Data were entered and analyzed using SPSS version 24.0. Mean ± Standard Deviation (SD) and Median (Inter Quartile Range, IQR) were computed for all quantitative variables. All the categorical variables were presented as frequencies and percentages. Chi-square test was applied to assess association between various categorical variables. Mann-Whitney U-test was applied to assess difference in quantitative variables between the continuous variables. P-value <0.05 was considered statistically significant.

## RESULTS

### Healthcare Workers (HCW) Result

A total of 71 participants were enrolled in the study and their demographics are given in Table-I. When asked about participants’ perception regarding “if women require pain relief in labor”, then regardless of age groups, qualification and designation, the majority (87.3%) felt that women need pain relief during labor (p=0.396, 0.580, 0.212 respectively).

**Table I:** Characteristics of healthcare workers.

Demographics	
Age (year), n=71	
Med (IQR)	26 (24 - 30.5)
*Age*	
16 – 24	21 (29.6)
25 – 30	31 (43.7)
> 31	17 (23.9)
Not reported	2 (2.8)
Total	71 (100)
*Gender*	
Male	14 (19.7)
Female	56 (78.9)
Not reported	1 (1.4)
*Educational qualification*	
Inter and Below	9 (12.7)
Graduation and above	56 (78.9)
Not reported	6 (8.5)
Total	71 (100)
*Current position*	
Doctors	15 (21.1)
Nurse/Midwife	41 (57.7)
Technician	13 (18.3)
Not reported	2 (2.8)
Total	71 (100)

On questioning about methods available for pain relief in labor (multiple answers were acceptable), 74.6% (n=53) reported both regional anesthesia and pain killers, 25.4%(n=18) reported general anesthesia, followed by local anaesthesia 16.9% (n=12) and counseling 21.1% (n=15) only. Moreover, participants reported systemic opioids with adjuvants as the most common means of pain relief during labor (36.6% n=26), followed by systemic opioids only (31%, n=22) and regional anesthesia (23.9%, n=17) (Table-II).

**Table II:** Questions asked and answers for methods for pain relief.

Available methods for pain relief	n (%)
Regional anesthesia	53 (74.6)
Pain killer	50 (70.4)
General anesthesia	18 (25.4)
Counseling	15 (21.1)
Local anesthesia	12 (16.9)
Inhalational	9 (12.7)
Support	4 (5.6)
Other	3 (4.2)
*Method of pain relief used most commonly in labor*	
Systemic opioids	22 (31)
Systemic opioids with adjuvants	26 (36.6)
Regional anesthesia	17 (23.9)
DK	4 (5.6)
Missing information	2 (2.8)
*Perception of participants regarding pain relief and counseling of pregnant women*	
*Do you think women need pain relief during labor?*	
Strongly disagree	4 (5.6)
Disagree	3 (4.2)
Agree	40 (56.3)
Strongly agree	22 (31)
Missing information	2 (2.8)
*Thinking about the pain relief in labor?*	
Should be practiced and has no adverse effect	31 (43.7)
It prolongs the labor	21 (29.6)
Affects the fetus adversely	10 (14.1)
Increase the risk of C-section	8 (11.3)
Other	1 (1.4)
*Pregnant female be counseled for labor analgesia*	
Antenatal	46 (64.8)
At the time of admission	14 (19.7)
At the time of labor	11 (15.5)

On enquiring about their (HCW) perception regarding pain relief in labor, almost half (43.7%) felt that pain relief should be practiced and has no adverse effect. Some believed it prolongs labor (29.6%), others (14.1%) replied that it affects the fetus adversely, and 11.3% reported increased risk of Cesarean section. No significant association of age groups, qualification and designation was found with perception of pain relief in labor (p=0.277, 0.534, 0.164 respectively).

With regards to timing of analgesia counselling, two-thirds of HCWs said that pregnant women should be approached at the antenatal stage, 19.7% at the time of admission and 15.5% at the time of labor. However, none were in favor of not counseling the women for labor analgesia. Moreover, results showed that higher proportion of technicians felt that counseling should be done at the time of admission and of labor as compared to doctors, nurses and mid-wives (p=0.001) (Table-II).

### Patient Results

### General

A total of 1005 pregnant women, median age (IQR) of 26 (24-29) years were included in this study. Of these, 135 (13.4%) were illiterate, 213 (21.2%) had primary to secondary education, 651 (64.8%) had higher than secondary school education (6 had missing data regarding education). Almost all the illiterate patients reported that they can neither read nor write.

Most women (n=698, 69.5%) were multigravida. Of these, the majority (n=298, 42.7%) reported that they had labor pain for four to 12 hours in previous deliveries. Some had less than four hours of pain (n=108, 15.5%) and others 13 to 18 hours of pain (n=198, 28.4%) previously. However, 67 (9.6%) reported labor pain of more than 18 hours in their last delivery. Additionally, when asked about severity, most multigravida women reported severe (n=349, 50.0%) or moderate (n=298, 42.7%) pain in their last delivery. Only 35 (5.0%) reported no pain.

On enquiring about delivery fears, most (n=573, 57.0%) reported they were moderately afraid of delivery complications, followed by great fear in 39% (n=392) and only 30 (3.0%) reported no fears at all. More than half of respondents (n=538, 52.5%) reported excessive fear to labor pain followed by moderate fear (n=442, 44%) and no fear to labor pain (n=34, 3.4%).

There was a significant association of fear of delivery complications and fear of labor pain in the multigravida group (325 (46.6%) and 349 (50%) for moderate and severe pain respectively, p-value 0.037). A very small percentage of primigravida reported no fear of delivery complication or fear of labor pain 2.6%(n = 8) and 3.3% (n = 10) respectively (Table-III).

**Table III:** Association of parity with fear of delivery complication and labor pain.

	Parity		P-value

	Primigravida^a^ n(%)	Multigravida^b^ n(%)	
*Fear of delivery complication*			
No fear	8(2.6)	22(3.2)	0.000**^†^
Moderate fear	106(35.1)^b^	466(67.3)	
Great fear	188(62.3)^b^	204(29.5)	
Total	302(100)	692(100)	
*Fear of labor pain*			
No fear	10(3.3)	24(3.4)	0.037*^†^
Moderate fear	116(38)^b^	325(46.6)	
Great fear	179(58.7)^b^	349(50)	
Total	305(100)	698(100)	

* P-value<0.05,

** P-value<0.0001,

† Pearson Chi Square test

-For significant pair, the key of the category (a=Primigravida, b=Multigravida) appears in the superscript.

### Knowledge about labor analgesia

Out of the total respondents, only 280 (27.9%) were aware of labor analgesia. Amongst them most (n=178, 63.9%) reported doctors as source of information, followed by friends (n=63, 22.5%) and family (n=10, 3.9%). The remaining patients (n=29) did not respond.

### Willingness to get labor analgesia after getting knowledge about it

Respondents were given complete information regarding labor analgesia and asked if they are willing to receive this or not. The majority (85.2%) showed willingness to receive labor analgesia while 14.1% reported non-willingness and 0.7% did not respond. Patients willing to get labor analgesia were older as compared to those unwilling (p<0.0001).

Of those who did not show willingness for epidural (108 in total), 35 (25%) reasoned labor pain a natural process that did not need intervention, 40 (28.6%) believed that motherhood required endurance of these pains, whereas 33 (23.6%) were not confident about the procedure.

It was found that women who reported fear of labor pain showed interest in receiving labor analgesia (p<0.0001). Out of these women, most were ready to pay for it (82.5%), whereas, 17.2% refused to pay. In addition, unwilling women were asked if they would consider free-of-cost labor analgesia. Despite this option, 102 (72.5%) of them showed continued reluctance towards receiving the intervention and 36 (25.4%) agreed to it. The remainder (n=3, 2.1%) did not respond.

## DISCUSSION

Less than half of participant HCWs were of the opinion that labor analgesia should be practiced. Meanwhile, amongst patients, despite most being multigravida, only 27.9% of the total were aware of labor analgesia. Upon learning about labor analgesia, 85.2% were willing to get analgesia for labor pain. This suggests a wide gap in knowledge and perceptions of labor analgesia in both HCWs and patients.

The findings of patient knowledge regarding labor analgesia are consistent with other studies from the subcontinent.^8^,^9^ The data from HCWs is difficult to assess as no such data is available in Pakistan. A number of studies from LMICs in Africa have demonstrated low level of knowledge and similar results.^1^ The knowledge of HCWs was audited in a study performed in India in 2004^10^ and a lack of exposure to training and knowledge of labor analgesia was seen.

To the best of our knowledge, this is the first study where knowledge and attitude of HCWs were surveyed for labor analgesia in Pakistan. The HCWs knowledge regarding labor analgesia and its different modalities is lacking, specifically in nurses and technicians. Yet HCWs coming in direct contact with patients can be a source of valuable information to pregnant women and their relatives regarding labor and labor analgesia. There appears to be lack in training regarding labor analgesia among healthcare workers this finding is similar to other studies.^11^,^12^

Less than a third (n=280, 27.9%) of patients were their options for pain relief. After getting information, most elderly patients were willing to receive analgesia for duration of labor. These findings are similar to those found in similar studies from other LMICs.^4^-^6^ Those patients willing to receive labor analgesia did not consider cost of service as an issue, this was surprising keeping in view that the data was collected at a charity hospital. We did not come across any study that reviewed the costing of service as a factor for labor analgesia in a LMIC (market prices of epidural-kits was shared participating patients during interview).

### Limitations of the study

It was conducted in consecutive patients coming to the antenatal clinic in one full month and in a single center located in a low-income area. The results may vary if the study was done in a private setting and/or if patients with higher level of education or higher socio-economic status were interviewed. A multi-center study of both patients and healthcare workers will give a much through picture of the knowledge gaps and attitude towards labor analgesia.

## CONCLUSION

This study shows a large gap in knowledge of HCWs as well as patients regarding labor analgesia. All HCWs in obstetric units should have appropriate level of knowledge to counsel patients regarding labor and labor analgesia. The majority of patents have insufficient knowledge of it but once informed, are willing to avail the service, irrespective of cost. Hence, information about it should be available to all patients.

### Author`s Contribution

**MA:** Patient Data collection and manuscript.

**SFS:** Conception, design of study and editing manuscript, takes the responsibility for integrity of research.

**AK:** Healthcare worker data collection and tables.

**NG:** Statistical analysis of healthcare worker data.
